# Lower incidence of CMV infection and acute rejections with valganciclovir prophylaxis in lung transplant recipients

**DOI:** 10.1186/1471-2334-13-582

**Published:** 2013-12-10

**Authors:** Inger Johansson, Gunnar Mårtensson, Ulla Nyström, Salmir Nasic, Rune Andersson

**Affiliations:** 1 Department of Infectious Diseases, Sahlgrenska Academy, University of Gothenburg, Gothenburg, Sweden; 2 Department of Respiratory Medicine, Sahlgrenska Academy, University of Gothenburg, Gothenburg, Sweden; 3 Transplant Institute, Sahlgrenska University Hospital, Gothenburg, Sweden; 4 Research and Development Centre, Skaraborg Hospital, Skövde, Sweden

**Keywords:** Bronchiolitis obliterans syndrome, Cytomegalovirus, Ganciclovir, Lung transplantation, Survival rates, Valganciclovir

## Abstract

**Background:**

Cytomegalovirus (CMV) is the most common opportunistic infection following lung transplantation. CMV replication in the lung allograft is described as accelerating the development of bronchiolitis obliterans syndrome (BOS). Finding a strategy to prevent CMV infection is an important issue.

**Methods:**

We performed a retrospective, single-centre study of 114 lung transplant recipients (LTRs) who underwent lung transplantation from January 2001 to December 2006. In a smaller cohort of 88 CMV seropositive (R+) LTRs, three months of valganciclovir prophylaxis (2004-2006) was compared to three months of oral ganciclovir (2001-2003) with respect to the incidence of CMV infection/disease, the severity of CMV disease, acute rejection, BOS-free 4 year survival and 4 year survival. In the whole group of 114 LTRs the impact of CMV infection on long-term survival (BOS free 4 year survival and 6 year survival) was assessed.

**Results:**

For the cohort of 88 CMV seropositive LTRs, the incidence of CMV infection/disease at one year was lower in the valganciclovir group compared to the ganciclovir group (24% vs. 54%, p = 0.003). There was a tendency towards reduced CMV disease, from 33% to 20% and a significant lower incidence of asymptomatic CMV infection (22% vs. 4%, p = 0.005). A lower incidence of acute rejection was observed in the valganciclovir group. However, there was no significant difference between the two groups in BOS free 4 year survival and 4 year survival.

For the entire group of 114 LTRs, BOS-free 4 year survival for recipients with CMV disease was (32%, p = 0.005) and among those with asymptomatic CMV infection (36%, p = 0.061) as compared with patients without CMV infection (69%). Six year survival was lower among patients with CMV disease, (64%, p = 0.042) and asymptomatic CMV infection (55%, p = 0.018) than patients without CMV infection (84%).

**Conclusions:**

A lower incidence of CMV infection/disease and acute rejections was observed with valganciclovir (3 months) when compared to oral ganciclovir (3 months). The long-term impact of CMV infection/disease was significant for BOS-free survival and survival.

## Background

Cytomegalovirus (CMV) is the most clinically significant opportunistic infection that can occur following lung transplantation (LTx). The reported incidence of CMV infection/disease ranges from 38% to 75% in lung transplant recipients (LTRs) in the absence of any prophylaxis [1]. In addition, CMV infections can have indirect effects; such as allograft rejection, diminished graft and patient survival as well as a predisposition for opportunistic infections and malignancies [2,3]. Finding strategies to prevent CMV infection/disease is a major challenge following lung transplantation. In a previous study of 187 LTRs we reported that oral ganciclovir (GCV) prophylaxis for 3 months delayed the onset and reduced the severity of CMV disease when compared to intravenous (IV) ganciclovir for 4 weeks [4]. Today, IV GCV followed by VGCV or VGCV alone are the most common prophylaxis strategies [5]. However, there is no consensus on the optimal duration of prophylaxis for LTRs. Valganciclovir is a prodrug of ganciclovir with greater bioavailability (60%) than oral GCV (6%) and oral GCV 1000 mg t.i.d.is equivalent to VGCV 450 mg daily [6].

The main cause of long-term morbidity and mortality in LTRs is bronchiolitis obliterans syndrome (BOS) that is present in 49% of recipients 5 years after lung transplantation and at 10 years the rate reaches 75% [7]. BOS is a chronic allograft dysfunction/chronic rejection that is defined as a progressive airflow obstruction not explained by acute rejection, infection or other confounding complication [8]. CMV replication in lung allograft is described as accelerating the development of BOS [9-13].

The aims of this study were to compare 3 months of CMV prophylaxis using oral ganciclovir with 3 months of valganciclovir with respect to incidence and severity of CMV disease and to study the impact of CMV infection/disease on long-term outcomes/survival.

## Methods

### Patient population and study design

Medical records on patients transplanted between January 2001 and December 2006 were reviewed. During the study period 128 lung transplantations were performed. Of these, 117 transplantations in 114 patients were selected for inclusion. Six patients who died within 30 days of transplantation and five re-transplantations were excluded. CMV infection, acute cellular rejection (AR) and BOS related to the initial lung transplantation were evaluated. Clinical episodes of CMV infection and AR at 12 months were evaluated. BOS development was followed annually. Follow-up on all patients was complete on January 20, 2011.

The primary goal of the study was to compare 3 months of prophylaxis with VGCV to GCV in both the short and long term. To exclude the influence of CMV serostatus, only the 88 patients with R + were included in this first cohort. All patients received VGCV or GCV; which was the standard care for CMV prophylaxis to R + .

A secondary goal was to assess the impact of CMV infection/disease (regardless of prophylaxis) on the development of BOS and survival. In this part of the study, both R + and R- patients were included to get a larger group (i.e. the second cohort).

This research was approved by the local Ethical Committee at Gothenburg University Hospital, Gothenburg, Sweden and it follows the applicable standards set by the Declaration of Helsinki; reference number Ö 393-01.

### Data collection

To identify CMV episodes; clinical parameters such as fever, cough, dyspnoea, hypoxemia and other clinical signs or symptoms mentioned in a clinical record were recorded as well as results from transbronchial biopsy (TBB) and bronchoalveolar lavage (BAL) to detect CMV infection, (ie.histopathology with CMV inclusion bodies and IHC analyses with monoclonal antibodies against CMV). Analyses of CMV replication by PCR were registered if it was performed, (i.e.in our study quantitative analysis of CMV DNA in serum). Results from TBB were collected to exclude or verify acute rejections.

Data from regular appointments was also collected to detect CMV infection (for details see the section on postoperative follow-up). Spirometry with FEV_1,_ to annually evaluate BOS development.

### Immunosuppression

Induction therapy used antithymocyte globulin (ATG) with an initial dose of 2.0 mg × 1 followed by 1.5 mg × 1. The doses were based on daily CD3-positive T lymphocyte cell counts and were given until the concentration of cyclosporine was 350 ng/ml, (in most cases 2-4 doses of ATG). Together with ATG, 500 mg of methylprednisolone was given twice the day of transplantation; following surgery 125 mg of methylprednisolone was administered every 8 hours for a total of 3 doses.

All patients received triple immunosuppressive therapy that used cyclosporine (CsA), mycophenolate mofetil (MMF) and prednisone. The dose of CsA was monitored so as to give an initial trough level of 350 ng/ml (0-3 months). This level was tapered to 300 ng/ml (3-6 months) and lastly tapered to 200-150 ng/ml at 6 months. MMF was given in the dose of 1.5 g × 2/day which was then reduced down to 1 g × 2/day after 3 months. Oral prednisone was initially given at the rate of 0.3 mg/kg/day (0-3 months). It was then tapered to 0.2 mg/kg/day (3-6 months) and finally tapered to 0.1 mg/kg/day at 6 months. If MMF was not tolerated it was switched to azathioprine (Aza) at the rate of 2 mg/kg/day. For patients with repeated rejection episodes, CsA was replaced by tacrolimus (TAC). The dose of TAC was monitored to maintain an initial trough level of 25-15 ng/ml (0-3 months). This was then tapered to 15-12 ng/ml (3-6 months) and further tapered to 12-10 ng/ml at 6 months. No or minimal changes were made in the immunosuppressive regimes during 2001-2003 when oral GCV was used as prophylaxis and from 2004 to 2006 when VGCV was used as prophylaxis.

### CMV prophylaxis

All R + patients were given oral GCV 1000 mg three times a day for 3 months. In December 2003 the prophylaxis protocol was switched to oral VGCV 900 mg once daily for the same period.

All D+/R- patients were given oral GCV 1000 mg three times a day for three months together with CMVIG (Megalotect®) 50 E/kg/day on days 0, 7, 14, 35, 56 and 77 after transplantation. In December 2003 the prophylaxis was switched to oral VGCV 900 mg once daily for 6 months; plus 6 doses of CMVIG over the same time frame as described in the last sentence.

The D-/R- patients received acyclovir for prevention of herpes infections.

IV GCV (except to R-) was used if a patient was not able to take oral medication. Doses of all drugs were adjusted for renal function.

### CMV treatment

The treatment for CMV disease and asymptomatic CMV infection verified by a lung biopsy was IV GCV 5 mg/kg twice daily for 10 to 21 days (sometimes longer).Patients with hypoxia also received polyclonal immunoglobulin (Gammagard®) at a dose of 0.5 g/kg every other day until improvement (maximum 5 doses). Patients who did not respond to GCV or who had severe bone marrow suppression were treated with foscarnet. A control biopsy was performed 4 weeks after the start of treatment. If needed, at least 2 more weeks of IV GCV 5 mg/kg twice daily was added. If CMV pneumonitis was not diagnosed, no further prophylaxis was given.

### Postoperative follow-up and monitoring

Regular appointments at the transplant unit, with clinical, radiological and pulmonary function evaluation and surveillance bronchoscopy using TBB/BAL were scheduled ½, 1, 2, 3, 4½, 6, 9, 12, 18 and 24 months after transplantation and then annually.

Pulmonary function tests consisted of spirometry (forced vital capacity - FVC), forced expiratory volume in 1 second (FEV_1_), measurements of lung volume (total lung capacity - TLC), functional residual capacity (FRC), residual volume (RV), carbon monoxide - CO uptake (i.e. transfer factor/diffusing capacity) and the single breath N2 test. TBB and BAL were also used if infection or rejection was suspected.TBB and BAL were repeated 4 weeks after episodes of acute rejection.

TBB samples were used for histopathologic examination including evaluation of acute or chronic rejection, the opportunistic fungus Pneumocystis jiroveci and CMV infection. Evaluation of CMV infection was performed using immunohistochemistry (IHC) with monoclonal antibodies against CMV.

BAL samples were cultured for fungi and bacteria, including Legionella and mycobacteria,

BAL samples were examined microscopically for CMV inclusion bodies, Pneumocystis jiroveci, other fungi and mycobacteria, throughout the study period.

From 2001 to 2006 all follow-ups were carried out using the same protocol.

### Definitions

#### Asymptomatic CMV infection

Detection of viral inclusion bodies (‘owl’s eye’) or a positive immunohistochemistry (IHC) in TBB and/or BAL together with parenchymal diffuse or perivascular inflammation or CMV DNA detected in serum, but with no clinical symptoms.

#### Mild CMV pneumonitis

In addition to CMV infection, at least 1 of the following signs and/or symptoms: subfebrility (≥ 37.5–37.9°C) ≥ 1 day, dyspnoea, cough or decreased FEV_1_ and/or decreased CO diffusion.

#### Moderate CMV pneumonitis

In addition to CMV infection, at least 2 of the following signs and/or symptoms: fever ≥ 38°C for ≥ 1 day, dyspnoea, cough or decreased FEV_1_ and/or decreased CO diffusion or white blood cell count (WBC) < 4 × 10^9^/l or platelet count < 100 × 10^9^/l.

#### Severe CMV pneumonitis

In addition to CMV infection, treatment with continuous positive airway pressure (CPAP) or ventilator was required, or had CMV-related mortality.

#### Prolonged CMV pneumonitis

CMV was found in TBB and/or BAL after 4 weeks or more.

#### Gastrointestinal CMV disease

Clinical symptoms such as nausea, vomiting, epigastric pain, diarrhoea, abdominal cramps, together with an endoscopically proven ulcer having microscopic mucosal lesions. If CMV is detected by a positive IHC of the biopsy, we define it as a ‘proven’ gastrointestinal CMV disease. If CMV DNA only was found in serum or the biopsy we call it ‘probable’ gastro-intestinal CMV disease. If a patient had severe symptoms from the gastrointestinal tract and pos CMV DNA in serum, but no endoscopy was performed, we call it ‘possible’ gastrointestinal CMV disease.

#### CMV syndrome

Fever, leucopenia or thrombocytopenia together with CMV DNA in serum and no other cause of symptoms/signs identified.

TBB was routinely evaluated morphologically together with immunohistochemistry (IHC) using monoclonal antibodies to identify early and late CMV antigens (Avidin-Biotin Complex Method for IHC Detection). The same IHC has been used during the study period.

CMV DNA in serum was analyzed by quantitative real-time PCR.

These definitions are based on a modified version from a publication by Ljungman et al [14]. The following definitions; the severity of CMV pneumonitis, prolonged CMV pneumonitis and possible gastrointestinal CMV disease are not generally accepted. The authors have found the definitions useful when it’s necessary to describe the symptoms more clearly.

*Bronchiolitis obliterans syndrome* (*BOS*) is defined as a baseline of forced expiratory volume in 1 second (FEV_1)_ calculated as a mean of the two best measurements obtained at the 2, 3, 4.5, 6 and 9 month follow-up appointments at the transplant unit following transplantation. This baseline value is used for comparison with FEV_1_ values measured later and to calculate a patient’s BOS grade. BOS grade 1 has an FEV_1_ of 65-80%, BOS grade 2 has an FEV_1_ of 50%-65% and BOS grade 3 and FEV_1_ less than 50% of the baseline value.

*Obliterative bronchiolitis* (OB) is the histological correlate of chronic allograft rejection; a peribronchiolar infiltration of lymphocytes, leading to fibrous scaring in the bronchioles.

*Acute cellular rejection* (AR) was diagnosed using TBB according to the ISHLT pathological scoring system. (A1 = minimal AR, A2 = mild AR, A3 = moderate AR, A4 = severe AR).

*Cumulative acute rejection score* (CAR score) was defined as the cumulative grading of AR ≥ 1 (e.g. A1 + A1 + A2 = CAR score of 4) [15].

*CAR score /TBB* are defined as an individual’s CAR score divided by their measurable TBB [11].

### Statistical analyses

**Figure 1:**
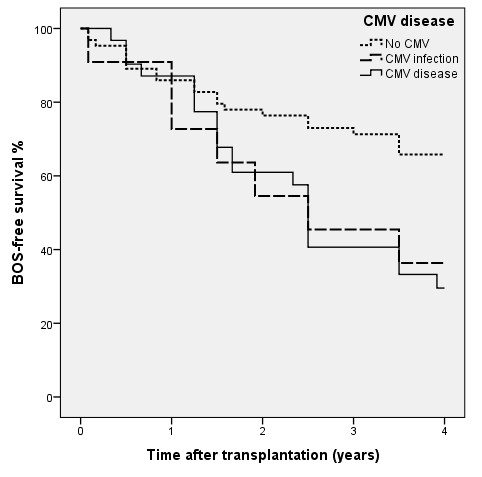
**BOS-free survival in 107 lung transplant recipients related to CMV disease.** No CMV infection (n = 65), Asymptomatic CMV infection (n = 11), CMV disease (n = 31). BOS-free 4 year survival for patients with CMV disease was 32%, (p = 0.005), for asymptomatic CMV infection 36%, (p = 0.061) as compared with patients without CMV infection (69%).

**Figure 2:**
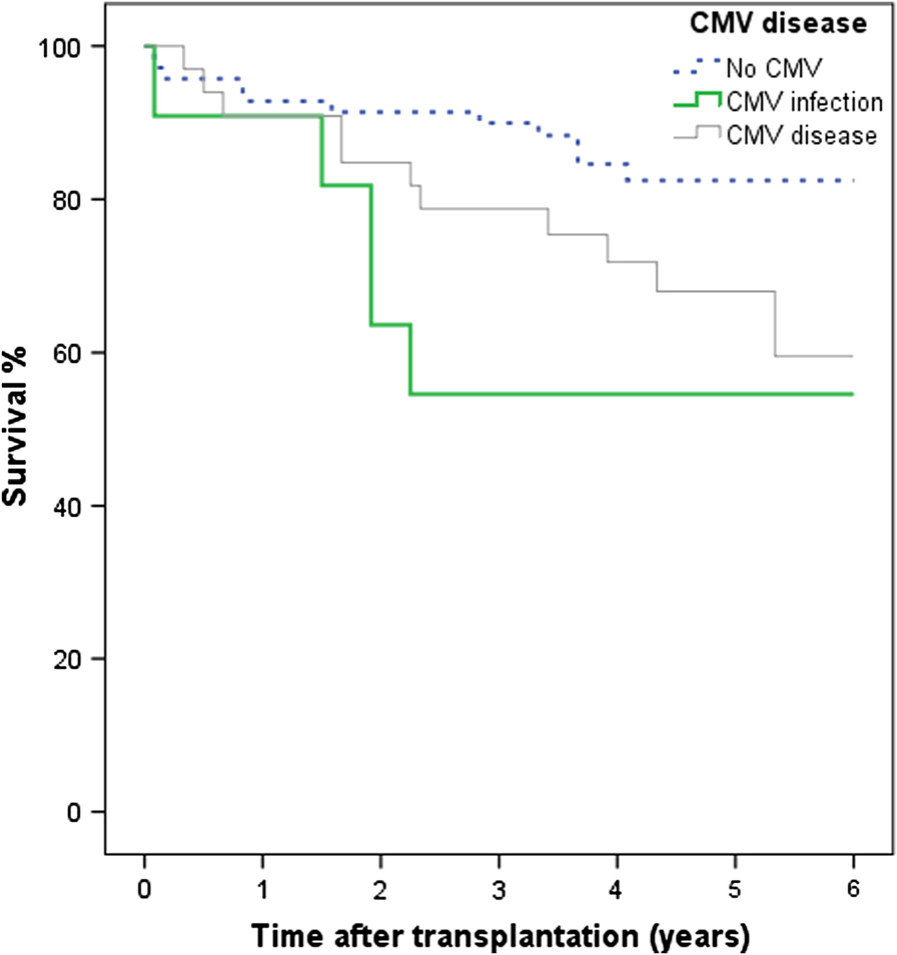
**Survival in 114 lung transplant recipients related to CMV disease.** No CMV infection (n = 70), Asymptomatic CMV infection (n = 11), CMV disease (n = 33). Six year survival was lower among patients with CMV disease, (64%, p = 0.042) and asymptomatic CMV infection (55%, p = 0.018) than patients without CMV infection (84%).

Descriptive statistics were calculated for continuous variables, frequencies and proportions for categorical variables. The Chi-square test was used to compare proportions and occurrences between the groups. When the median was assessed, the statistical comparisons used the Mann-Whitney test. Confidence intervals were calculated using a normality approximation algorithm. The survival analysis in Figures 1 and 2 used the Kaplan–Meier procedure. Comparisons of survival distributions between different categories were made using the log rank test. For the data used in Figure 1, patients without BOS information were excluded; except for those who died within one year of transplantation. Statistical significance was set at the 5% level i.e. p-value <0.05. Data was analyzed using SPSS version 20.

## Results

The demographics for the 114 LTRs are presented in Table 1. There were 72 woman (63%). The mean age of all members of the group was 49 years. The most frequent pre transplant diagnoses were chronic obstructive pulmonary disease (COPD) of 38%, idiopathic pulmonary fibrosis (IPF) of 20% and alpha-1 antitrypsin deficiency with emphysema (A1AT) of 18%. Single lung transplantation was performed with 70% of the recipients.

**Table 1:** Demographics for the 114 lung transplant recipients in the study

**Characteristics**	**N = 114**
LTx, years	2001-2006
Recipient Age years, mean (range)	49 (10-68)
Female (%)	63
Donor Age years, mean (range)	42 (5-70)
Female (%)	55
CMV serostatus, n (%)	D**+**/R**+**: 66 (58)
	D**-/**R**+**: 22 (19)
	D**+**/R-: 17 (15)
	D**-**/R -: 9 (8)
Tx type n (%)	
-Single	80 (70)
-Double	31 (27)
-Heart and lung	3 (3)
Pre-transplant diagnosis (%)	
-COPD	38
-IPF	20
-A1AT	18
-CF	6
-PAH + PH	6
-GVHD	3.5
-Scleroderma	3.5
-Eisenmenger syndrome	2
-Others	3

*R* = recipient, *D* = donor, *COPD* = chronic obstructive pulmonary disease, *CF* = Cystic fibrosis, *A1AT* = alpha-1 antitrypsin deficiency with emphysema, *IPF* = idiopathic pulmonary fibrosis, *PAH* = pulmonary arterial hypertension, *PH* = pulmonary hypertension, *GVHD* = graft-versus-host disease.

### Oral GCV versus VGCV for 3 months in 88 R + recipients

The demographics for the 88 LTRs are presented in Table 2. The demographics were equal concerning age and diagnosis; single lung transplantation was slightly more common in the GCV group (78% vs. 71%).

**Table 2:** Demographics of the 88 CMV seropositive (R+) lung transplant recipients

**Characteristics**	**Oral ganciclovir 3 months**	**Valganciclovir 3 months**
	**(n = 37)**	**(n = 51)**
LTx, years	2001-2003	2004-2006
Recipient age years, mean (range)	49 (15-64)	52 (13-67)
Female (%)	68	63
Donor age years, mean (range)	40 (11-61)	42 (5-70)
Female (%)	51	57
CMV + (%)	81	71
Tx type (%)		
-Single	78	71
-Double	19	29
-Heart and lung	3	0
Pre-transplant diagnosis (%)		
-COPD	41	37
-A1AT	24	16
-IPF	19	25
-CF	5	2
-PAH	3	6
-Eisenmenger syndrome	3	0
-Sarcoidosis	3	0
-GVHD	3	4
-Scleroderma	0	6
-Pulmonary embolism	0	4

None of the differences were statistically significant.

*COPD* = chronic obstructive pulmonary disease, *CF* = Cystic fibrosis, *A1AT* = alpha-1antitrypsin deficiency with emphysema, *IPF* = idiopathic pulmonary fibrosis, *PAH* = pulmonary arterial hypertension, *GVHD* = graft-versus-host disease.

Follow up for CMV infection was 12 months. The incidence of CMV infection/disease was lower in the VGCV prophylaxis group than in the GCV group (24% vs. 54%, p = 0.003). There was also a trend towards lower incidence of CMV disease in the VGCV group (20% vs. 33%, p = 0.17), see Table 3.

**Table 3:** Episodes of CMV infection or disease during the first 12 months in 88 CMV- seropositive (R+) lung transplant recipients

**CMV infection or disease**	**Oral ganciclovir 3 months**	**Valganciclovir 3 months**	**p-value**
**(n %)**	**n = 37**	**n = 51**	
LTx, years	2001-2003	2004-2006	
No CMV, n (%)	17 (46)	39 (76)	
CMV infection/disease, n (%)	20 (54)	12 (24)	0.003
CMV disease -Total n (%)	12 (33)	10 (20)	0.170
-Severe	0	2 (4)	0.262
-Moderate	8 (22)	5 (10)	
-Mild	4 (11)	3 (6)	
Asymptomatic CMV inf., n (%)	8 (22)	2 (4)	0.005
Prolonged CMV episode, n (%)	1 (3)	2 (4)	0.756
Gastrointestinal CMV inf., n (%)	2 (5)	2 (4)	0.741
Ganciclovir resistance, n (%)	0	1 (2)	0.392
Onset of CMV inf/disease, days,			0.188
Median, mean	174,163	154,136^a^	
(range)	(100-270)	(23-201^a^)	

^a^Onset of CMV infection was 62 days after transplantation for one patient who probably had a GCV resistant virus. Two patients had no or inadequate CMV prophylaxis owing to renal failure. Onset of CMV infection for these two patients was 23 days and 32 days after transplantation. For the remaining individuals in the VGCV group the onset of CMV infection/disease was on average 168 days after transplantation (range: 147 – 201 days).

In our study, 6% (5/88) had CMV infection diagnosed by CMV-DNA in serum. In the GCV group 2 patients were diagnosed with CMV DNA in serum (one with CMV syndrome and the other with possible gastrointestinal CMV disease). In the VGCV group three patients were diagnosed with CMV DNA in serum (one with an asymptomatic CMV infection and two with CMV syndrome).

From the medical records reviewed we found that 4 individuals in the R + cohort were diagnosed by a physician as having gastrointestinal CMV disease. Three had severe upper gastrointestinal tract symptoms and pos CMV DNA in serum; the fourth had lower gastrointestinal tract symptoms plus verified CMV pneumonitis.

With three exceptions, the onset of CMV infection in the VGCV group was between 147 and 201 days post-transplantation. Two of the three had no or inadequate CMV prophylaxis owing to renal failure (onset of CMV infection was for these two 23 and 32 days post-transplantation) and for the patient with a probable GCV resistant, the onset of CMV infection was at 62 days. Data for the length of CMV prophylaxis was missing for 2 of the persons in the GCV group and for 3 from the VGCV group; the remainder received prophylaxis for a median of 89 (GCV) or 90 (VGCV) days.

Acute rejections (AR) were studied for 12 months. AR was less frequent in the VGCV group during the entire first year, se Table 4. The CAR score divided by the number of evaluable TBBs and one or two treatable acute rejections (i.e. AR grade ≥ 2) were decreased in the VGCV group.

**Table 4:** Incidence of acute rejection (AR) at 3 and 12 months

	**Oral ganciclovir**	**Valganciclovir**	**p-value**
	**3 months (n = 37)**	**3 months (n = 51)**	
CAR score^a^, *median (quartiles)*			
3 months	2 (0 – 4)	0 (0 – 2)	0.02
12 months	3 (2 – 6)	1.5 (0 – 3)	0.002
CAR score/TBB^b^, *median (quartiles)*			
3 months	0.5 (0 - 1)	0 (0 - 0.5)	0.008
12 months	0.43 (0.2-0.67)	0.14 (0-0.5)	0.001
At least one episode of AR grade ≥ 1, *% (n)*			
3 months	68% (25/37)	44% (20/45)	0.03
12 months	84% (31/37)	62% (28/45)	0.02
At least one episode of AR grade ≥ 2, *% (n)*			
3 months	51% (19/37)	29% (13/45)	0.04
12 months	73% (27/37)	49% (22/45)	0.03
At least two episodes of AR grade ≥ 2, *% (n)*			
3 months	24% (9/37)	13% (6/45)	n.s
12 months	41% (15/37)	20% (9/45)	0.04
Number of TBB, *median (quartiles)*			
3 months	4 (3 – 4)	4 (3 – 4)	n.s
12 months	9 (8 – 9)	8 (6 – 8)	0.001

^a^Cumulative acute rejection score (CAR score) varied between 0 and 6 at 3 months and between 0 and 16 at 12 months.

^b^Cumulative acute rejection score (CAR score) divided by the number of evaluable transbronchial biopsies (TBB).

#### BOS-free survival and survival in R+

BOS-free 4 year survival in R + (both the GCV and VGCV groups) was 30% for patients with CMV disease and 67% for those without CMV infection (p = 0.018). There was no significant difference in long-term outcome (i.e. 4 year survival and BOS-free 4 year survival) between the GCV and VGCV prophylaxis groups.

#### BOS and OB in R+

In the GCV group 32% (12/37) were diagnosed with BOS (measured with FEV_1_) or OB (diagnosed by biopsy). Six of these patients died and one was re-transplanted owing to BOS.

In the VGCV group 24% (12/51) were diagnosed with BOS/OB. Two of these patients died and 2 were re-transplanted owing to BOS.

#### Mortality in R+

In the GCV group, a total of 27% (10/37) died within 4 years. Six of these individuals died of BOS. Two died of malignancies, one of infection (not CMV infection) and one of lung bleeding.

In the VGCV group 20%, (10/51) of the patients died within 4 years. Two died of BOS, two of malignancies and six patients died of infections. (One of them died owing to CMV disease).

### Episodes of CMV infection or disease during the first 12 months in 114 lung transplants recipient (R + and R-)

Demographics are shown in Table 1. Follow-up for CMV infection was 12 months.

The impact of CMV serostatus was essential for onset of CMV infection. The incidence of CMV infection/disease was for D+/R- 65%, for D+/R + 39% for D-/R + 27% and for D-/R- 11% (p = 0.03).

CMV disease was found in 29% (33/114) of the study participants. Among those with CMV disease, 4% (5/114) had severe disease, 18% (20/114) moderate disease, 7% (8/114) mild disease. Asymptomatic CMV infection was found in 10% (11/114) and no CMV infection was found in 61% (70/114) of the study group. In this cohort only 5% (6/114) were diagnosed with CMV DNA in serum.

Gastrointestinal (GI) CMV disease was seen in 8% (9/114) of the study group. Four patients from the high risk group (D+/R-) had proven GI CMV disease. One individual from the low risk group (D-/R-) suffered from a primary infection and had a proven GI CMV disease (as well as CMV pneumonitis). Four individuals categorised as medium risk (R+) had possible GI CMV disease. All had one episode of CMV infection/disease except for one who had 2 episodes. Prolonged CMV episodes were found in 9% (10/114) of the entire study group. In the high-risk group 35% (6/17) had a prolonged CMV episode.

### Long-term follow up in 114 lung transplant recipients (R + and R-) according to the development of CMV infection

#### BOS-free survival in the total group (R + and R-)

BOS-free 4 year survival (i.e. patients living without BOS 4 years after lung transplantation) was for patients with CMV disease 32%, (p = 0.005), for asymptomatic CMV infection 36%, (p = 0.061) as compared with patients without CMV infection, 69%, see Figure 1. BOS data was available for 107 of the 114 patients. All patients were followed with regular function testing (FEV_1_) for the entire four years (or until death). BOS-free survival was on the average 2.9 (95% CI; 2.6-3.2) years for the total group. Patients without CMV infection were free from BOS for 8.4 months longer than those with a CMV disease/infection. Patients with D+/R- had the shortest time until the detectable onset of BOS.

#### Survival in the total group (R + and R-)

Six year survival was lower among patients with CMV disease, (64%, p = 0.042) and asymptomatic CMV infection (55%, p = 0.018) as compared with patients without CMV infection (84%). Six year survival for the entire group of 114 patients was 75% see Figure 2. No patients were lost to follow-up tracking. Four of the 11 patients with asymptomatic CMV infection died early - 2 of malignancy, 2 of infection (not CMV infection). The deaths of 4 out of 11 may at least partially explain the apparently poor responses among the study participants with asymptomatic CMV infection.

#### GCV resistant CMV

Two patients were treated with foscarnet. One had a proven GCV resistance (mutation in the CMV UL 97 gene). The other had a rapid response after being switched from ganciclovir to foscarnet but no resistance test was performed. CMV serostatus for these two were D+/R- and D+/R + .

## Discussion

Oral prophylaxis with VGCV seemed to be more effective than GCV among the LTRs studied. A lower incidence of CMV infection/disease at one year was observed, 24% versus 54% (p < 0.003), mainly due to a reduction of asymptomatic CMV infection from 22% to 4%. Montforte et al performed a similar study comparing 120 days of VGCV to GCV prophylaxis in LTRs and they found a lower rate of CMV disease in the VGCV group, 16 versus 8%, but the difference did not reach statistical significance [16]. There was no difference in the rate of asymptomatic CMV infection. In their study regular monitoring by shell vial or CMV antigenemia assay was performed while in our study we monitored frequently with surveillance TBBs including IHC diagnosis. Only a few patients were diagnosed with CMV infection/disease solely on the basis of positive CMV DNA in serum. Monitoring of blood, today mostly done by molecular assays, might lead to earlier detection of asymptomatic CMV infection which enables treatment and prevention of CMV disease. Our extensive use of surveillance TBBs could also be a contributing factor to our higher rate of tissue-invasive CMV disease.

There was no difference between GCV and VGCV treatment regarding the time for onset of CMV infection/disease or the number of prolonged CMV episodes. The rate of gastrointestinal disease, 4-5%, was also similar in the groups. Gastrointestinal CMV may be underreported in our study since patients with verified CMV pneumonitis treated with IV GCV who at the same time had gastro-intestinal symptoms were not regularly undergoing an endoscopy.

Our centre uses ATG as induction therapy, which is supposed to give a higher rate of CMV disease. However, the monitoring with CD3-positive T-cells has made it possible to reduce ATG by 50% as compared to our previous fixed dose of ATG, why it now should only have limited effect on the rate of CMV disease. We found that CMV disease was still 20% at 1 year with VGCV prophylaxis for 3 months. It may be possible to further reduce this rate with a longer prophylaxis and frequent monitoring with quantitative CMV PCR after cessation of VGCV prophylaxis. CMV prophylaxis after lung transplantation varies widely between centres; most follow a regimen that has 3-6 months of prophylaxis with IV GCV or VGCV with or without CMVIG [5]. Recent studies recommend extending prophylaxis for LTRs [17-22]. Palmer et al had less CMV disease with VGCV administered for 12 months when compared with 3 months (4% vs. 32%, p < 0.001) [21].

In our study the frequency of ganciclovir resistance based on treatment failures when using IV ganciclovir was 2% (2/114). As we did not routinely use CMV DNA detected by PCR during the study period, GCV resistance may have been underreported. As only two patients needed treatment with foscarnet instead of ganciclovir it is also possible that regular monitoring with quantitative CMV PCR would not necessarily have meant that more recipients with GCV resistance would have been identified. The overall incidence of antiviral-resistant CMV in LTRs has been reported to be 6-9% and in high-risk groups (D+/R-) 10-27% [23,24].

We found a significant decrease in the rate of acute rejection in the VGCV group; at 3 and 12 months. Between 2001 and 2006 there were few if any changes in the immunosuppression protocol used by our centre and the significant reduction of acute rejections in the relatively small VGCV group is therefore an important observation. One explanation as to why the AR rate decreased could be that the significantly lower incidence of CMV infection/disease during the first year post transplant among those receiving the VGCV prophylaxis represented less inflammatory response in the transplanted lung(s); which then triggered fewer episodes of AR. Paraskeva et al observed a significantly lower incidence of acute rejection within the first 12 months when VGCV for 5 months was compared with GCV for 3 months when there was no difference in the immunosuppression protocols between the two groups [13]. Jaksch et al found that there was a non-significant positive trend towards a lower acute rejection score when D+/R- LTRs received 12 months of VGCV when compared with 3 months [20]. Cumulative acute rejection score (CAR) score is not used universally but many authors have found that it is a useful tool for measuring and assessing combined severity and frequency of rejection over time. CAR score and CAR score divided by the number of evaluable TBBs has been used elsewhere and reported in other studies [11,13,20,25].

We found a tendency towards lower BOS/OB with VGVV prophylaxis (24% vs. 32%). Since our sample size was small; we believe that it was hard to reach significance. Development of BOS is believed to be an ongoing immunological process triggered by various factors as frequency and severity of acute rejections, CMV infection, other infections, differences in HLA antigens between donor and recipient as well as other inflammatory processes [8]. CMV infections usually occur during the first year post transplantation, it’s therefore not surprising that when we compared BOS-free 4- year survival after transplantation in R+, we found no significant difference between the treatment regimens considering the limited study population.

The effect on development of BOS associated with CMV infection/disease as such could be regarded as the negative impact from CMV infection/disease is stronger than the difference in impact between the two drugs, it’s therefore not surprising that greater BOS development is noted for CMV infection/ disease as such compared to the effect caused by the difference of the drugs.

BOS-free 4 year survival for R + individuals was 30% for those with CMV disease and 67% for those without a CMV infection.

To better evaluate the impact of CMV on BOS progression we included R- to give a larger study group. We found that BOS-free 4 year survival for the total group was 55% and it was lower for those with CMV disease (32%) compared to the 69% rate for those without CMV infection (p = 0.005).

Our results support the idea that CMV infection is a risk factor for onset of BOS. Other studies have pointed out that CMV infection is one of the reasons for development of BOS [9,11-13,17,19,26] yet other studies did not find that CMV infection had any impact of the onset of BOS [27,28].

The survival after 6 years (in R + and R-) was 64% for those diagnosed as having CMV disease (p = 0.042), 55% for those with an asymptomatic CMV infection (p = 0.018) when compared with patients without CMV infection (84% survival).

The strength of our study is the long-term follow-up, verification of CMV diagnosis by using frequent consecutive surveillance TBBs (including IHC for CMV), and the uniform evaluation and treatment process achieved because Sahlgrenska is a single centre. FEV_1_ was followed for the entire four years. No or minimal changes were made in the immunosuppressive regimes during the period 2001-2006. The definition of CMV was identical throughout the study period.

A limitation is that different CMV prophylaxis drugs were studied during different periods of time. Another is that quantitative CMV DNA in serum was not used regularly during the study period. Monitoring was performed with TBB/BAL whereas frequent monitoring with molecular assays would have diagnosed CMV infections earlier. Confounding factors with the potential to reduce the CMV infection rate may have included more frequent use of tacrolimus towards the end of the study period - although we do not believe that this affected our findings. Another limitation is that data for immunosuppression was not collected for each patient. In the VGCV group a few more double lung (DL) transplants were included – this may have brought about a lowered development of BOS and improved survival.

## Conclusions

We observed a lower incidence of CMV infection/disease and acute cellular rejection during VGCV prophylaxis when compared with oral GCV – both being administrated for 3 months. CMV disease/infection was 24% in the VGCG group and this rate needs to be reduced further. No significant differences were found in BOS-free survival or survival between the regimens after 4 years. The impact of CMV infection/disease in the total group (regardless of CMV serostatus and prophylaxis) was significant for BOS-free 4 year survival and 6 year survival.

## Competing interests

The authors declare that they have no competing interests.

## Authors’ contributions

IJ participated in the design of the study, reviewed the medical records, evaluated CMV infection, the severity of CMV disease and drafted the manuscript. GM reviewed the medical records, evaluated FEV_1_ and BOS grade. SN is a statistician and was responsible for the statistical analysis. UN collected critical data, including donor CMV serostatus and date of death, and assisted in collection of the other clinical data. RA and GM participated in the design of the study and reviewed the manuscript. All authors have read and approved the final manuscript.

## Pre-publication history

The pre-publication history for this paper can be accessed here:

http://www.biomedcentral.com/1471-2334/13/582/prepub
